# TMEM211 Promotes Tumor Progression and Metastasis in Colon Cancer

**DOI:** 10.3390/cimb45060287

**Published:** 2023-05-24

**Authors:** Yung-Fu Chang, Hsing-Hsang Wang, Chih-Wen Shu, Wei-Lun Tsai, Cheng-Hsin Lee, Chun-Lin Chen, Pei-Feng Liu

**Affiliations:** 1 Department of Biomedical Science and Environmental Biology, Kaohsiung Medical University, Kaohsiung 80708, Taiwan; m795003@kmu.edu.tw (Y.-F.C.); austinwang680999@gmail.com (H.-H.W.); r980084@kmu.edu.tw (C.-H.L.); 2 Translational Research Center of Neuromuscular Diseases, Kaohsiung Medical University Hospital, Kaohsiung 80708, Taiwan; 3 Department of Medical Research, Kaohsiung Medical University Hospital, Kaohsiung 80708, Taiwan; 4 Institute of BioPharmaceutical Sciences, National Sun Yat-sen University, Kaohsiung 80424, Taiwan; cwshu@g-mail.nsysu.edu.tw; 5 Department of Internal Medicine, Kaohsiung Veterans General Hospital, Kaohsiung 81362, Taiwan; wltsai@vghks.gov.tw; 6 Department of Biological Sciences, National Sun Yat-sen University, Kaohsiung 80424, Taiwan; chunlinchen@mail.nsysu.edu.tw; 7 Center for Cancer Research, Kaohsiung Medical University, Kaohsiung 80708, Taiwan

**Keywords:** transmembrane protein 211, colon cancer, prognosis, epithelial–mesenchymal transition, signaling pathways

## Abstract

Colon cancer is the third most important cancer type, leading to a remarkable number of deaths, indicating the necessity of new biomarkers and therapeutic targets for colon cancer patients. Several transmembrane proteins (TMEMs) are associated with tumor progression and cancer malignancy. However, the clinical significance and biological roles of TMEM211 in cancer, especially in colon cancer, are still unknown. In this study, we found that TMEM211 was highly expressed in tumor tissues and the increased TMEM211 was associated with poor prognosis in colon cancer patients from The Cancer Genome Atlas (TCGA) database. We also showed that abilities regarding migration and invasion were reduced in TMEM211-silenced colon cancer cells (HCT116 and DLD-1). Moreover, TMEM211-silenced colon cancer cells showed decreased levels of Twist1, N-cadherin, Snail and Slug but increased levels of E-cadherin. Levels of phosphorylated ERK, AKT and RelA (NF-κB p65) were also decreased in TMEM211-silenced colon cancer cells. Our findings indicate that TMEM211 regulates epithelial–mesenchymal transition for metastasis through coactivating the ERK, AKT and NF-κB signaling pathways, which might provide a potential prognostic biomarker or therapeutic target for colon cancer patients in the future.

## 1. Introduction

Colon cancer is the third most important cancer type, with an estimated 1,918,030 new cancer cases and 609,360 cancer deaths for the United States in 2022 [1,2]. There were approximately 1,148,515 new cases and 576,858 deaths globally caused by colon cancer in 2020 [3]. There are several possibilities for the first-line treatment of colorectal cancer [4]. The reasons causing ineffective treatment include colon cancer stem cells in cancer relapse, chemoresistance and sophisticated molecular mechanisms [5]. Due to the fact that many people are suffering from colon cancer worldwide, the development of new diagnostic bio-markers and therapeutic agents for colon cancer are in urgent demand [4].

The family of transmembrane proteins (TMEMs) contains at least one putative transmembrane segment spanning biological membranes of the plasma and organelles [6]. Several of them have unknown functions, but others belong to fibroblast growth factor receptor proteins or ion channel proteins [7]. TMEMs are involved in several diseases, such as panic disorder, neurodegenerative disease, anxiety, nerve-injury-induced pain behaviors, and erythrocytosis [8,9,10,11,12]. TMEMs are also involved in several biological functions such as cell proliferation, invasion/migration and drug resistance in several cancers [6]. For example, TMEM45B, TMEM48, and TMEM16A are essential for cancer metastatic processes such as invasion and epithelial–mesenchymal transition (EMT), intravasation and extravasation [7]. TMEM48 was reported to be involved in the invasion of lung cancer cells [7]. TMEM45B knockdown in lung cancer cell lines A549 and NCI-H1975 reduced the invasive potential of these cells. In vitro studies showed that TMEM16A could enhance cell proliferation and the migration abilities of cancer cells. Therefore, TMEMs were shown to provide prognostic biomarkers for different tumors. For example, TMEM45B was shown to be overexpressed in clinical samples of gastric cancer. RNA interference of TMEM45B inhibited the migration and invasion activities in human gastric adenocarcinoma HGC-27 cells, and TMEM45B expression was associated with poor prognosis in lung cancer patients [7]. TMEM48 and TMEM97 are potential prognostic biomarkers for lung cancer [6]. The TMEM48 expression level was higher in tumor tissues compared to healthy tissues in non-small-cell lung carcinoma (NSCLC) patients. The overexpression of TMEM48 was associated with lymph node metastasis, increased tumor size, poor prognosis and short survival of NSCLC [6]. The expression level of TMEM97 was higher in tumor tissue compared to normal tissues of NSCLC patients. Moreover, TMEM97 protein level was correlated with poor tumor differentiation and a shorter survival in patients with NSCLC [6]. The TMEM16A protein was found to be highly expressed in several tumor tissues including glioma, oral, head and neck squamous cell carcinoma (HNSCC), esophageal, lung, gastrointestinal and breast cancers [13]. A high expression level of TMEM45A was found in the breast cancer and cervical lesions and correlated with a lower overall survival for the patient, indicating TMEM45A as a potential biomarker for the aggressiveness of breast cancer and cervical lesions [14]. Therefore, TMEMs participate in cancer processing.

A few TMEMs regulate colon cancer processing. For example, inhibiting the expression of TMEM16A, a calcium-activated chloride channel, resulted in delayed cell cycle progression in colorectal SW620 cells. TMEM16A is composed of eight transmembrane segment regions with the N- and C-termini facing the cytoplasm and a reentrant loop located between TM5 and TM6, possibly forming the pore region. Knocked-down TMEM16A inhibited cell proliferation, migration and invasion in colorectal cancer SW620 cells. The knockdown of TMEM16A was accompanied by decreased expression of p-MEK, p-ERK1/2 and cyclin D1. Therefore, TMEM16A regulates the growth and metastasis of colorectal cancer, possibly by regulating the MAPK pathway [13]. TMEM255 was screened for differentially methylated genes and differentially expressed genes, using the TCGA database to obtain gene methylation and gene expression difference in colon cancer samples [15]. TMEM211 shows different expression levels between KRAS G12 mutated and wild-type colorectal cancer [16]. However, the particular roles of TMEM211 in colon cancer remain unknown as yet.

TMEM211 is also called LHFPL7 (LHFPL Tetraspan Subfamily Member 7) and BA9F11.1. Its gene is located on chromosome 22q11.23 of humans to encode a protein with 200 amino acids. The TMEM211 protein contains four predicted alpha-helical transmembrane regions at amino acids 5–27, 68–88, 113–133 and 150–170. However, the biological function of TMEM211 was never identified. Therefore, we study the potential functions of TMEM211 in this research article.

EMT is involved in the initial migratory and invasive steps of cancer progression [17,18,19]. It causes polarity loss of epithelial cells, the detachment of cells from the basement, and allows tumor-initiation and metastases of cancer cells [20,21]. EMT occurs during tumorigenesis accomplished by decreased E-cadherin expression for separating cell–cell adhesion [22,23]. The function of E-cadherin is to prevent tumor cells dissociating from one another and prevent their migration to other tissues. In addition, N-cadherin is upregulated in the process of EMT, for promoting angiogenesis [24,25]. Mesenchymal cells are more motile, less polarized and highly expresses N-cadherin. Upregulated N-cadherin has been shown to promote invasion of epithelial-derived cancer cells. There are several transcriptional factors participating in the process of EMT called EMT-inducing transcriptional factors, such as Slug, Snail and Twist1. Snail promotes the acquisition of the migratory and invasive properties of EMT by regulating several transcriptional targets, resulting in tumor malignancy [26]. Slug reduces E-cadherin expression, increases lymph node metastasis and is associated with poor prognosis in various types of cancers [27]. SNAIL is a key moderator of tumor aggressiveness and metastasis formation by the induction of the EMT program and the subsequent acquisition of stem-cell-like features [28]. In addition, SNAIL has EMT-independent oncogenic functions in cancer, and bypasses traditional oncogenic KRAS-induced senescence but blocks the retinoblastoma-controlled senescence pathway for cancer progression in pancreatic ductal adenocarcinomas. Twist1 induces EMT by decreasing epithelial gene expression and increasing mesenchymal gene expression. It is also involved in tumor initiation, primary tumor growth and metastasis. Twist1 induces N-cadherin expression for invasiveness in oral tongue squamous cell carcinoma [29]. TMEM 16A regulates the expression of E-cadherin [30]. TMEM16A silencing reduces the expression of E-cadherin in gastric cancer cells, AGS and BGC-823. Overexpressed TMEM229A reduces N-cadherin, while TMEM229A knockdown exerts the opposite effect in NSCLC cells, demonstrating that TMEM229A suppresses cell migration and the invasion of NSCLC cells by regulating the EMT phenotype [31]. TMEM17 upregulates the expression of Snail in breast cancer cells, MCF-7 and MDA-MB-231 [32]. Overexpressed TMEM17 upregulates the Snail protein level, while TMEM17 knockdown exerts the opposite effects. However, the regulation of TMEM211 in EMT fostering metastasis in colon cancer has not been reported. 

TMEM regulates biological functions, possibly through different molecular mechanisms. The ERK/MAPK signaling pathway is involved in invasion and metastasis [33]. TMEM229A suppresses the progression of NSCLC through inactivating the ERK pathway [31]. The overexpression of TMEM229A reduces the expression of phosphorylatation levels of ERK, p-ERK, and ERK inhibitor (PD98059) partially suppresses this effect, indicating that the TMEM229A-inhibited tumor progression is partially mediated by inactivating the ERK signaling pathway in NSCLC. The PI3K/AKT signaling pathway phosphorylates a series of substrates to affect cellular migration [34]. TMEM63c increases the cell viability of human podocytes via Akt signaling [35]. The down-regulation of TMEM63c by siRNA decreases the ratio of p-Akt/Akt and cell viability, but increases cell apoptosis of human podocytes. In addition, the nuclear factor-kappa B (NF-κB) signaling pathway is also involved in metastasis, where NF-κB/p65 (RelA) regulates EMT in breast cancer cells [36]. Interestingly, several studies have shown that TMEMs activate ERK, AKT and NF-κB signaling pathways for tumor progression. For example, TMEM43 contributes to EGFR-induced NF-κB activation in epidermoid carcinoma cells [37]. The overexpression of TMEM43 induces NF-κB activation detected by the NF-κB reporter assay, while suppressing TMEM43 expression by shRNA impairs the NF-κB activation in HEK293T cells. TMEM119 facilitates invasion and migration by regulating the AKT signaling pathway in ovarian cancer [38]. However, it is still unknown through which signaling pathway TMEM211 regulates EMT in colon cancer. 

In this study, we compared TMEM211 expression of normal and tumor tissues and analyzed the association of TMEM211 expression with the prognosis in human colon cancer patients. We hypothesize that TMEM211 plays a role in the migration/invasion and expression levels of EMT markers/molecules regarding ERK, AKT and NF-κB signaling pathways, and investigated this using TMEM211-silenced colon cancer cells. Our results present the clinical significance and metastatic mechanisms of TMEM211 in colon cancer. These results indicate that TMEM211 could serve as a potential prognostic biomarker, as well as a therapeutic target in colon cancer.

## 2. Materials and Methods

### 2.1. Cell Culture

The normal colon cells (FHC) and three CRC cell lines of colorectal cancer cells (DLD-1, HCT-116 and SW620) were obtained from ATCC and grown on Corning tissue culture plates (Corning Incorporated, Corning, NY, USA) containing Dulbecco’s modified Eagle’s medium (DMEM) (Invitrogen-Gibco, Carlsbad, CA, USA) with 10% FBS (Biological Industries, Kibbutz Beit Haemek, Haifa, Israel), 100 U/mL penicillin and 100 μg/mL streptomycin (Invitrogen-Gibco, Carlsbad, CA, USA), at 37 °C in a humidified 5% CO_2_ atmosphere.

### 2.2. Transient Transfection

CRC cells were transfected with 10 nM scramble siRNA or siRNA against TMEM211 (Ambion/Thermo Fisher Scientific, Grand Island, NY, USA), using RNAiMax reagent (Invitrogen Life Technologies, Carlsbad, CA, USA) for 72 h.

### 2.3. Real-Time PCR (RT-PCR)

The RT-PCR was performed using the StepOnePlus Real Time PCR System (Applied Biosystems, Foster City, CA, USA) combined with the SYBR Green Master Mix reagent (Applied Biosystems). The nucleotide sequences of TMEM211 primer (Forward: 5′-GCCTCCTCCATGTATTATGGTG-3′; Reverse: 5′-CCTTGGACCTTGGTTGTGTG-3′) were used in this study. More detailed procedures were described in a previous study [39].

### 2.4. Cell Invasion and Migration 

Cell invasion was performed using 8 μm pore transwell inserts (Greiner Bio-One, Stroud, UK) coated with 0.5% Matrigel. The invasive cells on the bottom surface of the filter were fixed, stained and counted under a microscope. Cell migration was performed using Ibidi Culture-Inserts (IBIDI, Inc., Planegg, Germany) for the wound-healing assay. The migration distance of the cells was observed and quantified. The detailed protocol was described in a previous report [40].

### 2.5. Western Blotting

The proteins from cell lysates were separated by SDS-PAGE and then transferred onto a nitrocellulose membrane. The nitrocellulose membrane was incubated with primary antibodies including p-ERK1/2, ERK1/2, p-AKT, AKT, p-RelA/NFkB p65 (Ser536) or RelA/NFkB p65 (Cell Signaling Technology, Danvers, MA, USA) at 4 °C overnight, and then with the HRP-labeled secondary antibody (Cell Signaling Technology, Danvers, MA, USA). The chemiluminescence was developed with the ECL reagent and visualized using the Syngene GeneGnome XRQ chemiluminescence imaging system (GeneGnome XRQ, SYNGENE, Cambridge, UK).

### 2.6. Statistical Analysis

The transcriptome data of 286 colon cancer patients were obtained from The Cancer Genome Atlas (TCGA) database (https://cancergenome.nih.gov, accessed on 2 July 2020) and analyzed with the SPSS software (version 20.0, IBM-SPSS Inc., Chicago, IL, USA). The AJCC/UICC-TNM (The American Joint Committee on Cancer/Union for International Cancer Control/Tumor-Node-Metastasis) cancer staging system was used in this study (Table S1) [41]. The expression level of TMEM211 between normal and tumor tissues was compared using the Student’s t test. The survival analysis of colon cancer patients was performed using the Cox proportional hazards model and the Kaplan–Meier curve, as well as the log-rank test. The operating characteristic curve was used for distinguishing low and high expression of the TMEM211 gene.

## 3. Results 

### 3.1. Differential Expression of TMEM211 of Normal and Tumor Tissues of Colon Cancer Patients

**Figure 1:**
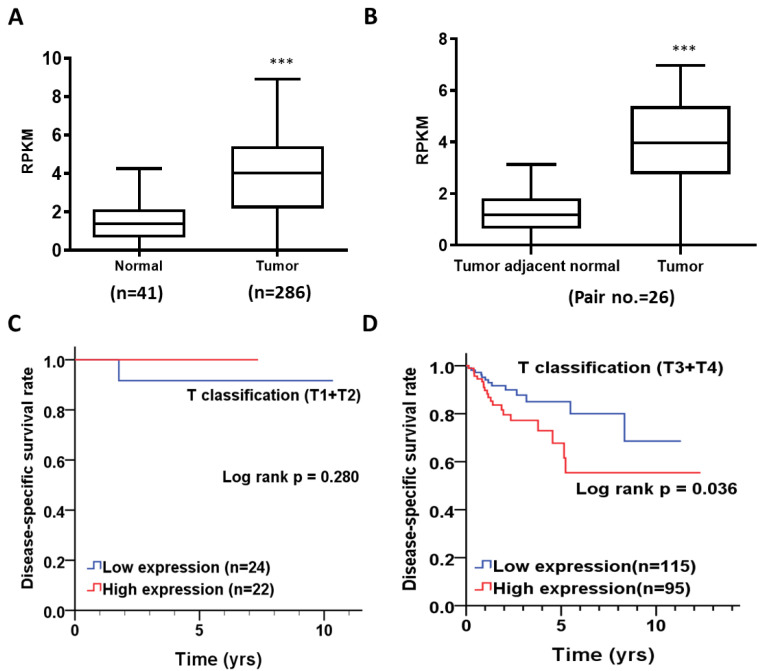
TMEM211 expression and its association with prognosis in colon cancer patients from the TCGA database. TMEM211 expressions were compared between (**A**) 41 normal and 286 tumor tissues of colon cancer patients or (**B**) corresponding adjacent normal and tumor tissues from 26 paired colon cancer patients. The correlation of TMEM211 expression with DSS in colon cancer patients stratified with (**C**) smaller tumor size (T1 + T2) and (**D**) larger tumor size (T3 + T4) was analyzed using the Kaplan–Meier method and the log-rank test (the *p*-value of significance is *** *p* < 0.001).

To investigate the expression level of TMEM211, we downloaded and analyzed the transcriptome data of colon cancer patients from the TCGA database. After analysis, we found that TMEM211 was highly expressed in tumor tissues (*n* = 286; mean ± SD = 3.82 ± 2.05, *p* < 0.001) compared to those in normal tissues (*n* = 41; mean ± SD = 1.43 ± 0.98, *p* < 0.001, Table 1; Figure 1A) from colon cancer patients. Moreover, expression levels of TMEM211 in tumor tissues (mean ± SD = 3.82 ± 1.86, *p* < 0.001, Table 2; Figure 1B) were also higher than those in the corresponding tumor adjacent normal (CTAN) tissues (mean ± SD = 1.34 ± 1.02, *p* < 0.001) from 26 paired colon cancer patients. These results demonstrate that TMEM211 is highly expressed in tumor tissues of colon cancer patients.

### 3.2. The Association of TMEM211 Expression with Prognosis in Colon Cancer Patients Stratified by Clinicopathological Outcomes 

We firstly analyzed the association of TMEM211 expression with clinicopathological outcomes, and found that the expression level of TMEM211 was not significantly different between female/male or young (≤70 y)/old (>70 y), having an early (I + II)/late (III + IV) pathological stage, having a small (T1 + T2)/large (T3 + T4) tumor size and having no (N0)/high risk (N1 + N2) lymph node metastasis in colon cancer patients (Table S2). We further investigated the association of TMEM211 expression with survival rate in colon cancer patients stratified with different clinicopathological outcomes. Our results showed that colon cancer patients with high TMEM211 expression had poor progression-free interval survival [PFIS, adjusted hazard ratio [33]: 1.91, 95% CI: 1.11–3.27, *p* = 0.019, Table 3] and disease-specific survival (DSS, AHR: 2.30, 95% CI: 1.15–4.60, *p* = 0.019, Table 3). Interestingly, compared to colon cancer patients with smaller tumor size (T1 + T2) (Table 4; Figure 1C), higher TMEM211 expression was associated with poor DSS in colon cancer patients with larger tumor size (T3 + T4) [AHR (95%) = 2.39, 95% CI: 1.18–4.85, *p* = 0.016, Table 4; log-rank test *p* = 0.036, Figure 1D). However, levels of TMEM211 expression were not associated with overall survival (Table S3), PFIS (Table S4) and disease-free interval survival (Table S5) in colon cancer patients stratified with clinicopathological stages or T classification. These results demonstrated that high TMEM211 expression was positively associated with shorter DSS, especially in colon cancer patients with large tumor size. 

### 3.3. The Involvement of TMEM211 in Migration/Invasion and EMT Marker Expression of Colon Cancer Cells

**Figure 2:**
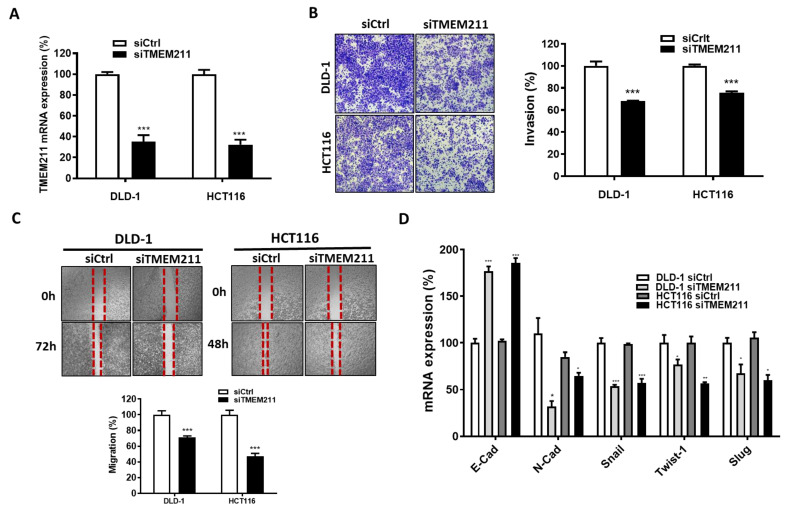
Invasion, migration and EMT marker expression in TMEM211-silenced colon cancer cells. (**A**) The efficiency of TMEM211 gene-silencing was analyzed by RT-PCR. (**B**) Cell invasion was measured by transwell migration assay. (**C**) Cell migration was measured by wound-healing assay. (**D**) The mRNA levels of E-cadherin (E-Cad), N-cadherin (N-Cad), Snail, Twist-1 and Slug were analyzed by RT-PCR. All HCT116 and DLD-1 cells were silenced with scramble siRNA (5 nM, siCtrl) or siRNAs against TMEM211 (5 nM, siTMEM211) for 72 h (*p*-values of significance are * *p* < 0.05; ** *p* < 0.01; *** *p* < 0.001).

It is reported that several TMEM members are involved in regulating EMT in various types of cancers. To firstly investigate whether TMEM211 is also involved in the migration and invasion of colon cancer cells, two colon cancer cells (HCT116 and DLD-1) with higher TMEM211 expression compared to normal colon FHC cells and other colorectal cancer cells (SW620) (Figure S1A) were silenced by siRNA against TMEM211 (Figure 2A). After silencing, TMEM211-silenced DLD-1 and HCT116 cells showed decreased abilities regarding invasion (Figure 2B) and migration (Figure 2C), compared to scramble cells. However, the migration of TMEM211-silenced SW620 cells with lower TMEM211 was not changed compared to that in scramble cells (Figure S1B). Thus, we further evaluated the effect of TMEM211 on the expression of EMT markers in DLD-1 and HCT116 cells. As the results show, the gene level of E-cadherin was significantly increased but the gene levels of N-cadherin, Snail, Twist1 and Slug were decreased in TMEM211-silenced cells (Figure 2D). Moreover, protein levels of N-cad and Snail were decreased but the protein level of E-cad was increased in TMEM211-silenced cells compared to that in scramble cells (Figure S2). Furthermore, colon cancer patients with high co-expression of TMEM211/EMT markers (Snail, Twist 1, Slug and N-cadherin) had poor DSS (Table 5). These results demonstrated that TMEM211 might be involved in metastasis by acting as regulators of EMT marker expression for a poor prognosis for colon cancer.

### 3.4. The Regulation of ERK, AKT and NF-κB Signaling Pathways by TMEM211 in Colon Cancer Cells 

**Figure 3:**
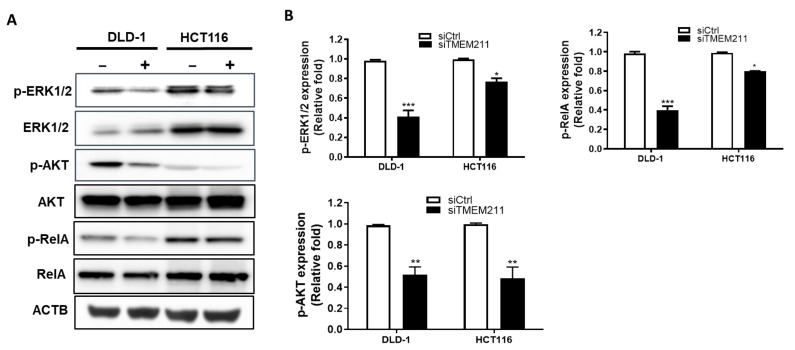
Expressions of molecules related to ERK, AKT and NF-κB signaling pathways in TMEM211-silenced colon cancer cells. (**A**) The levels of total and phosphorylated ERK1/2, AKT, RelA were analyzed using Western blotting. The β-actin (ACTB) was used as loading control. (**B**) The quantified levels of p-ERK1/2, p-AKT and p-RelA. All HCT116 and DLD-1 cells were silenced with scramble siRNA (5 nM, siCtrl) or siRNAs against TMEM211 (5 nM, siTMEM211) for 72 h. (*p*-values of significance are * *p* < 0.05; ** *p* < 0.01; *** *p* < 0.001).

It is known that several TMEMs are involved in metastasis through regulating the ERK, AKT, and NF-κB pathways [42]. To further study whether TMEM211 regulates the ERK, AKT and NF-κB pathways for metastasis in colon cancer cells, we compared the levels of ERK, AKT and NF-κB in scramble and TMEM211-silenced colon cancer cells. We found that all levels of phosphorylated ERK (p-ERK), p-AKT, and p-RelA were lower in TMEM211-silenced colon cancer cells (Figure 3) compared to those in scramble cells, especially in DLD-1 cells. Moreover, gene expression levels of RelA and Akt2 were slightly decreased in TMEM211-silenced cells compared to those in scramble cells (Figure S3). These results imply that TMEM211 might regulate ERK, AKT and NF-κB signaling pathways for metastasis in colon cancer cells. 

### 3.5. The Predicted Interaction of TMEM211 and Its Associated Proteins

**Figure 4:**
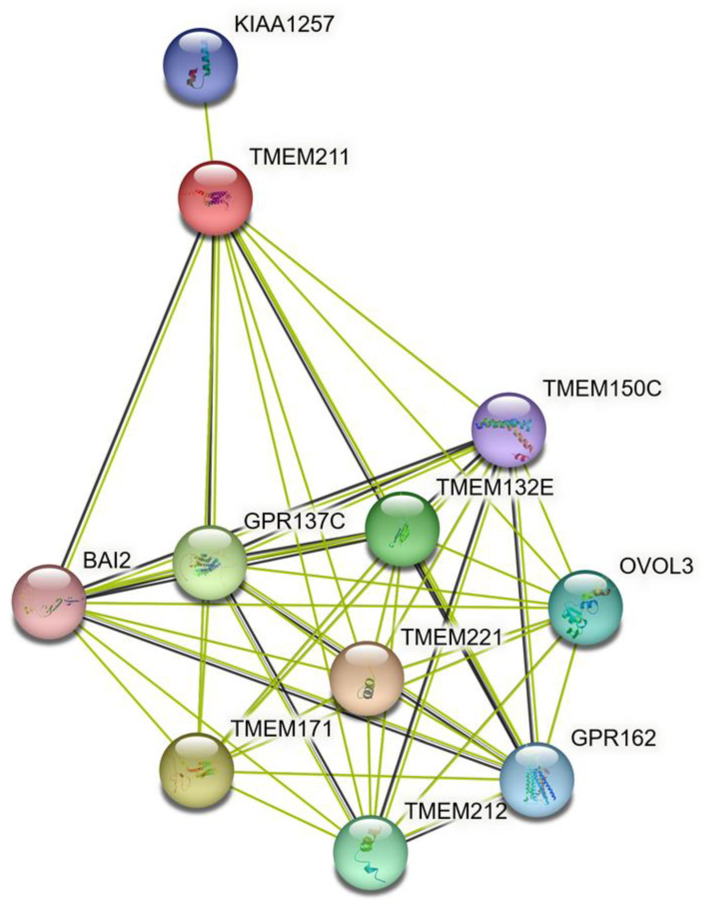
The interaction of TMEM211 and its associated proteins was analyzed using the STRING database (https://string-db.org, accessed on 2 July 2020).

In order to study the mechanism regulated by TMEM211, the associated proteins were predicted using the STRING database (https://string-db.org, accessed on 2 July 2020). The results showed that TMEM211 may associate with proteins including KIAA1257, BAI2, GPR137C, GPR162, and OVOL3, as well as other TMEMs, such as TMEM150C, TMEM132E, TMEM221, TMEM171 and TMEM212 (Figure 4). However, the association and the functions of interacting proteins need to be further explored. 

## 4. Discussion

TMEMs are involved in tumor progression, including the growth, chemoresistance and metastasis in various types of malignant tumors. Moreover, they were reported as potential biomarkers or therapeutic targets for several types of cancers [3,4]. Among TMEMs, TMEM211 is differently expressed between KRAS G12 mutated and wild-type colorectal cancer [16]. However, its role in colon cancer are still unclear. In this study, we firstly discovered that (1) TMEM211 was highly expressed in tumor tissues and upregulated TMEM211 was associated with shorter PFIS and DSS in colon cancer patients; (2) migration/invasion abilities were reduced and there was deregulated expression of EMT markers in TMEM211-silenced colon cancer cells; (3) the high co-expression of TMEM211/EMT markers in colon cancer cells indicated their correlation with poor DSS in colon cancer patients; and (4) TMEM211 should regulate ERK, AKT and NF-κB signaling pathways in colon cancer cells.

TMEMs were shown to have a very important clinical significance in several cancers. For example, high levels of TMEM180 were associated with poor survival in colorectal cancer patients of advanced pathological stages [43]. Both the hazard ratio of disease-free and specific survival in high-TMEM180 cases were higher than those in low-TMEM180 cases. Moreover, tumor-initiating activity was positively correlated with the level of TMEM180 expression in SW480 cells, indicating the important role of TMEM180 in colorectal cancer progression. High TMEM14A expression was positively associated with tumor size, tumor stage and recurrence of ovarian cancer cells [44]. TMEM14A was highly expressed in tumor tissues and correlated with poor prognosis in patients with ovarian cancer. Moreover, TMEM14A inhibited ovarian cancer cell apoptosis. Therefore, TMEM14A could provide both diagnostic and prognostic biomarkers for the early detection of ovarian cancer. TMEM17 expression was positively correlated with lymph node metastasis in breast cancer [32]. TMEM17 protein was upregulated in tumor tissues, especially in invasive breast cancer, compared to normal tissues. Its expression was closely related to the patient’s T-stage, advanced TNM stages and lymph node metastasis. Therefore, TMEM17 can promote the malignant progression of breast cancer. Moreover, a higher expression level of TMEM106C was closely related to the malignancy of hepatocellular carcinoma (HCC) [45]. The mRNA and protein level of TMEM106C were overexpressed in HCC tumor tissues and cell lines HepG2 and SMMC-7721. The cells treated with siTMEM106C suppressed the proliferation and metastasis abilities. The upregulation of TMEM106C was correlated with tumor stage and prognosis of HCC. Therefore, MEM106C contributes to malignance and poor prognosis of HCC. According to our results, high TMEM211 expression was associated with poor DSS in colon cancer patients having larger tumor sizes but not in patients with lymph node metastasis. Moreover, we found that TMEM211 promotes the invasion/migration of colon cancer cells. These results indicate that TMEM211 promotes local metastasis and causes larger tumor size. 

Some TMEMs were reported to regulate the process of invasion/migration in colorectal cancer (CRC) cells. For instance, TMEM16A was overexpressed and involved in the migration and invasion of metastatic brain tumors [46]. TMEM16A was found to be overexpressed in glioma, and helped to promote cell proliferation, migration and invasion through increased activity of the ERK and NF-κB signaling pathways. TMEM45B, TMEM48 and TMEM176A regulated the migration and invasion of many tumors [7]. TMEM45B siRNA inhibited the migration and invasion phenotype of gastric cancer HGC-27 cells. TMEM45B knockdown in lung cancer cell lines (A549 and H1299) also showed reduced invasive potential. TMEM48 knockdown showed decreased MMP-2 and MMP-9 expression in A549 and H1299 cells, indicating the role of TMEM48 in the invasion of lung cancer. However, TMEM176A negatively regulated cell invasion through reducing the expression of MMP-2/9 in different types of cancer such as glioma, esophageal, liver, and colorectal cancers. In our study, we found another member of TMEMs, TMEM211, to be involved in the invasion/migration and EMT marker regulation of CRC cells (HCT116 and DLD-1). These findings imply that several TMEMs are simultaneously involved in the invasion/migration of CRC.

TMEMs regulate tumor progression by different molecular signaling pathways, such as ERK and AKT. It is known that TMEM16A depletion suppresses the invasion in CRC cells, accompanied by the dysregulation of p-MEK and p-ERK1/2 expression [13]. TMEM16A shRNA decreased the expression of phosphorylation of MEK and ERK1/2 but did not affect the total MEK or ERK1/2 expression levels in high-metastatic-potential SW620 cells, and these results indicated that TMEM16A regulated the growth, migration and invasion of metastatic CRC cells through the MEK/ERK signaling pathway. TMEM43 promotes migration and invasion through the PRPF3/RAP2B/ERK axis in pancreatic cancer [47]. TMEM43 knockdown reduced RAP2B and p-ERK expression levels, compared to the corresponding control cells in MIAPaCa-2, SW1990, and Capan-2 cells. However, TMEM43 overexpression increased the levels of RAP2B and p-ERK in TMEM43-silenced MIAPaCa-2 cells compared to control cells. These data demonstrated that TMEM43 promoted pancreatic cancer progression through regulating the RAP2B/ERK axis. TMEM176A suppresses the progression of pancreatic cancer cells by inhibiting ERK signaling [48]. Overexpression of TMEM176A suppressed proliferation, invasion, and migration, but induced apoptosis in Capan-1 and PANC-1 cells. In addition, overexpression of TMEM176A resulted in ERK dephosphorylation, indicating that TMEM176A inhibits the ERK pathway. These results suggested that TMEM176A regulated pancreatic cancer progression through modulating the ERK pathways. Moreover, overexpressing TMEM229A inhibits the migration and invasion of NSCLC through reducing the expression levels of p-ERK and p-AKT [31]. Low expression of TMEM229A was associated with a poor prognosis. Overexpression of TMEM229A inhibited cell proliferation, migration and invasion, while TMEM229A knockdown had the opposite effect in NSCLC cells (A549 and H23 cells). Overexpressing TMEM229A reduced the levels of p-ERK and p-AKT, and this effect was partially suppressed by the ERK inhibitor, PD98059, in A549 and H23 cells. These results demonstrated that inhibition of cell proliferation, migration and invasion by TMEM229A were partially mediated by inactivating the ERK signaling pathway. Moreover, TMEM17 upregulated p-AKT for invasion and migration in breast cancer cells [32]. TMEM16A regulates the growth and metastasis of colorectal cancer, possibly by regulating the MAPK pathway [13]. Our result also showed that TMEM211 regulates colon cancer processing through the MAPK pathway. On the other hand, TMEM17 was upregulated in invasive cells compared to adjacent normal breast duct glandular epithelial cells. The overexpression of TMEM17 increased the levels of p-AKT, while TMEM17 knockdown had the opposite effect in breast cancer MCF-7 and MDA-MB-231 cells. Therefore, TMEM17 promotes the malignant progression of breast cancer cells by activating the AKT signaling pathway of breast cancer. TMEM116 promotes cell migration and invasion, via the regulation of the PDK1-AKT-FOXO3A pathway in lung cancer [49]. TMEM116 is highly expressed in NSCLC tissues and cell lines. Knockdown TMEM116 reduced cell proliferation, migration and invasiveness of A549 cells. Knockdown TMEM116 also reduced the protein level of PDK1, p-AKT and FOXO3A of lung cancer A549 cells. Therefore, TMEM116 promotes cancer development via the PDK1/AKT/FOXO3A signaling pathway. Furthermore, NF-κB is an important pathway in colorectal cancer progression [50]. However, there have been no articles describing the effect of TMEM211 on NF-κB in cancer, so far. Our result showed that TMEM211 could co-activate p-ERK, p-AKT and NF-κB.

To further confirm whether TMEM211 co-activates the ERK, AKT and NF-κB signaling pathways, TMEM211-silenced cells with re-expression of TMEM211 will be the essential complementary assay for verifying the activation of ERK, AKT and NF-κB signaling pathways, which require more work to verify. Moreover, TMEM211 downstream unknown mediators involved in activating the ERK, AKT and NF-κB signaling pathways will also need to be investigated.

Moreover, several studies showed that TMEMs are involved in the regulation of extracellular markers such as metalloproteinases. For example, TMEM196 silencing in lung cancer cells and mice resulted in the upregulation of MMP2 and MMP7 [51]. TMEM196 inhibited tumor metastasis and the progression of lung cancer in vitro and in vivo. TMEM196 overexpression suppressed the expression of MMP2, and of MMP7 in lung cancer cells, whereas TMEM196 knockdown had the opposite effect. The expression levels of MMP2, and MMP7 were decreased in lung tissue in the TMEM196 knockout mouse, indicating the relationship between TMEM and MMPs. TMEM229A overexpression reduced MMP2 expression, indicating that EMT was suppressed [31]. TMEM229A expression was downregulated in NSCLC tissues and in several cell lines, such as A549, H23, 95D, H226 and H1975. TMEM229A overexpression increased MMP2 expression, while TMEM229A knockdown exerted the opposite effect. Our analyzed data showed that colon cancer patients with the high co-expression of TMEM211/MMP2 or TMEM211/MMP9 had poor DSS (Table S6). Moreover, we found that the activity of MMP9 analyzed using zymography was slightly decreased in TMEM211-silenced cells compared to those in scramble cells (Figure S4), suggesting that TMEM211 might regulate MMP2 and MMP9 for metastasis in colon cancer.

Furthermore, we found that there is an interactive network between TMEM211 and other molecules, including GPR137C, GPR162, BAI2, KIAA1257, OVOL3, TMEM150C, TMEM132E, TMEM221, TMEM171, and TMEM212, according to the STRING analysis (Figure 4). One study has shown that different TMEMs are simultaneously presented in one type of cancer, HNSCC [52]. This is consistent with our analyzed data (Figure S2) showing that TMEM211 might correlate with other TMEMs for tumor progression in colon cancer. This analysis will help us to further understand possible molecular effects as mechanisms of TMEM211 affecting metastasis in colon cancer.

## 5. Conclusions

We firstly report here that TMEM211 promotes cancer progression and EMT for metastasis in colon cancer through coactivating the ERK, AKT and NF-κB signaling pathways, which implies that TMEM211 might be a potential prognostic biomarker and target for the treatment of colon cancer patients in the future.

## Figures and Tables

**Table 1:** The comparison of TMEM211 expression between normal and tumor tissues in colon cancer patients from TCGA database.

TMEM211 Expression	Normal (n = 41)		Tumor (n = 286)		*p* Value *
	Mean ± SD	Median	Mean ± SD	MEDIAN	
	1.43b ± 0.98	1.3842	3.82 ± 2.05	4.0342	<0.001

Abbreviations: SD, standard deviation. * *p* values were estimated by student’s *t*-test.

**Table 2:** The comparison of TMEM211 expression between corresponding tumor adjacent normal and tumor tissues in paired colon cancer patients from TCGA database.

TMEM211 Expression	Pair No.	Tumor Adjacent Normal		Tumor		*p* Value *
		Mean ± SD	Median	Mean ± SD	Median	
	26	1.34 ± 1.02	1.1866	3.82 ± 1.86	3.9703	<0.001

Abbreviations: Pair No, pair numbers of colon cancer patients; SD, standard deviation. * *p*-values were estimated by Paired *t*-test.

**Table 3:** The association of TMEM211 expression with different survival in colon cancer patients from TCGA database.

Variable	ROC	No. (%)	CHR (95% CI)	*p* Value *	AHR (95% CI)	*p* Value ^†^
Overall survival						
TMEM211	Low	213 (78.6)	1.00		1.00	
	High	58 (21.4)	1.44 (0.84–2.46)	0.187	1.69 (0.98–2.91)	0.059
Progression-free interval survival						
TMEM211	Low	89 (32.8)	1.00		1.00	
	High	182 (67.2)	1.72 (1.00–2.95)	0.047	1.91 (1.11–3.27)	0.019
Disease-specific survival						
TMEM211	Low	139 (54.3)	1.00		1.00	
	High	117 (45.7)	1.81 (0.91–3.59)	0.090	2.30 (1.15–4.60)	0.019
Disease-free interval survival						
TMEM211	Low	27 (28.4)	1.00		1.00	
	High	68 (71.6)	6.19 (0.81–47.41)	0.079	6.31 (0.82–48.47)	0.076

Abbreviations: ROC, operator characteristic curve for low and high expression; CHR, crude hazard ratio; CI, confidence interval; AHR, adjusted hazard ratio. * *p* values were estimated by Cox’s regression. ^†^ *p* values were adjusted for AJCC pathological stage (stage III + IV vs. stage I + II) by multivariate Cox’s regression.

**Table 4:** The association of TMEM211 expression with disease-specific survival in colon cancer patients from TCGA database.

Variable	ROC	No. (%)	CHR (95% CI)	*p* Value *	AHR (95% CI)	*p* Value ^†^
Sex						
Female	Low	69 (59.0)	1		1	
	High	48 (41.0)	0.72 (0.22–2.39)	0.591	0.75 (0.23–2.51)	0.645 ^a^
Male	Low	70 (50.4)			1	
	High	69 (49.6)	3.70 (1.34–10.24)	0.012	5.51 (1.89–16.09)	0.002 ^a^
Age, yrs						
≤70	Low	84 (53.8)	1		1	
	High	72 (46.2)	0.96 (0.40–2.31)	0.920	1.32 (0.54–3.23)	0.546 ^a^
>70	Low	55 (55.0)	1		1	
	High	45 (45.0)	4.25 (1.18–15.23)	0.026	4.95 (1.35–18.19)	0.016 ^a^
AJCC pathological stage						
I, II	Low	76 (52.8)	1			
	High	68 (47.2)	2.16 (0.40–11.83)	0.374	ND	ND
III, IV	Low	63 (56.3)	1			
	High	49 (43.8)	2.20 (1.03–4.69)	0.041	ND	ND
T classification						
T1, T2	Low	24 (52.2)	1		1	
	High	22 (47.8)	0.013 (0.00–135,544.68)	0.598	0.01 (0.00–139,945.54)	0.598 ^b^
T3, T4	Low	115 (54.8)	1		1	
	High	95 (45.2)	2.08 (1.03–4.20)	0.04	2.39 (1.18–4.85)	0.016 ^b^
N classification						
N0	Low	79 (53.0)	1		1	
	High	70 (47.0)	3.31 (0.67–16.41)	0.143	3.31 (0.67–16.41)	0.143 ^c^
N1, N2	Low	60 (56.1)	1		1	
	High	47 (43.9)	1.90 (0.87–4.14)	0.109	1.91 (0.88–4.18)	0.104 ^c^

Abbreviations: ROC, operator characteristic curve for low and high expression; CHR, crude hazard ratio; CI, confidence interval; AHR, adjusted hazard ratio. * *p* values were estimated by univariate Cox’s regression. ^†^ *p* values were estimated by multivariate Cox’s regression. ^a^ Adjusted for AJCC pathological stage (stage III + IV vs. stage I + II). ^b^ Adjusted for N classification (N1, N2 vs. N0). ^c^ Adjusted for T classification (T3, T4 vs. T1 + T2). ND: Non-detectable.

**Table 5:** The association of TMEM211/EMT marker co-expression with disease-specific survival in colon cancer patients from TCGA database.

Variable	ROC	No. (%)	CHR (95% CI)	*p* Value *	AHR (95% CI)	*p* Value ^†^
TMEM211	Low	139 (54.3)	1.00		1.00	
	High	117 (45.7)	1.81 (0.91–3.59)	0.09	2.30 (1.15–4.60)	0.019 ^a^
TWIST1	Low	144 (56.3)	1.00		1.00	
	High	112(43.8)	1.86(0.94–3.67)	0.075	1.69 (0.86–3.35)	0.130 ^a^
TMEM211 (L), Twist1 (L)		83 (32.4)	1.00		1.00	
TMEM211 (H), Twist1 (L)		61 (23.8)	1.14 (0.53–2.45)	0.732	2.23 (0.79–6.28)	0.129 ^b^
TMEM211 (L), Twist1 (H)		56 (21.9)	1.20 (0.54–2.65)	0.657	2.33 (0.81–6.75)	0.119 ^b^
TMEM211 (H), Twist1 (H)		56 (21.9)	1.89 (0.92–3.90)	0.083	3.25 (1.19–8.85)	0.021 ^b^
Snail	Low	91 (35.5)	1.00		1.00	
	High	165 (64.5)	3.50 (1.36–9.05)	0.010	2.97 (1.15–7.68)	0.025 ^a^
TMEM211 (L), Snail (L)		44 (17.2)	1.00		1.00	
TMEM211 (H), Snail (L)		47 (18.4)	0.55 (0.19–1.56)	0.258	3.68 (0.41–32.98)	0.244 ^b^
TMEM211 (L), Snail (H)		95 (37.1)	1.02 (0.51–2.05)	0.951	6.20 (0.81–47.40)	0.079 ^b^
TMEM211 (H), Snail (H)		70 (27.3)	2.76 (1.40–5.46)	0.003	11.84 (1.57–89.41)	0.017 ^b^
Slug	Low	108 (42.2)	1.00		1.00	
	High	148 (57.8)	2.39 (1.11–5.13)	0.025	2.40 (1.12–5.14)	0.025 ^a^
TMEM211 (L), Slug (L)		57 (22.3)	1.00		1.00	
TMEM211 (H), Slug (L)		51 (19.9)	0.81 (0.34–1.97)	0.646	2.44 (0.61–9.77)	0.208 ^b^
TMEM211 (L), Slug (H)		82 (32.0)	1.09 (0.53–2.24)	0.812	3.06 (0.85–10.98)	0.087 ^b^
TMEM211 (H), Slug (H)		66 (25.8)	2.32 (1.17–4.61)	0.017	5.11 (1.47–17.86)	0.011 ^b^
N-cad	Low	119 (46.5)	1.00		1.00	
	High	137 (53.5)	2.67 (1.27–5.60)	0.010	2.37 (1.12–4.99)	0.024 ^a^
TMEM211 (L), N-cad (L)		62 (24.2)	1.00		1.00	
TMEM211 (H), N-cad (L)		57 (22.3)	0.87 (0.38–1.99)	0.736	3.02 (0.78–11.72)	0.110 ^b^
TMEM211 (L), N-cad (H)		77 (30.1)	1.33 (0.65–2.73)	0.438	4.16 (1.15–14.96)	0.029 ^b^
TMEM211 (H), N-cad (H)		60 (23.4)	2.32 (1.16–4.65)	0.018	6.17 (1.75–21.80)	0.005 ^b^

Abbreviations: ROC, operator characteristic curve for low and high expression; CHR, crude hazard ratio; CI, confidence interval; AHR, adjusted hazard ratio; N-cad, N-cadherin; L: low expression; H: high expression. * *p* values were estimated by Cox’s regression. ^†^ *p* values were estimated by multivariate Cox’s regression. ^a^ *p* values were adjusted for cell differentiation (moderate + poor vs. well) and AJCC pathological stage (stage III + IV vs. stage I + II) by multivariate Cox’s regression. ^b^ *p* values were adjusted for group comparison by multivariate Cox’s regression.

## Data Availability

Not applicable.

## Supplementary Materials

The following supporting information can be downloaded at https://www.mdpi.com/article/10.3390/cimb45060287/s1, Figure S1: TMEM211 expression and migration in normal colon and different colorectal cancer cells. Figure S2: The protein levels of E-cad, N-cad and Snail in TMEM211-silenced DLD-1 cells were analyzed using Western blotting. Figure S3: The gene expression levels of RelA and Akt2 in TMEM211-silenced DLD-1 cells were analyzed using RT-PCR. Figure S4: The MMP9 activity of TMEM211-silenced DLD-1 cells was analyzed using zymography. Table S1: AJCC/UICC-TNM and clinical staging for colon cancer patients. Table S2: The association of TMEM211 expression with clinicopathologic outcomes in colon cancer patients from the TCGA database. Table S3: The association of TMEM211 expression with overall survival of colon cancer patients from TCGA database. Table S4: The association of TMEM211 expression with progression-free interval survival in colon cancer patients from the TCGA database. Table S5: The association of TMEM211 expression with disease-free interval survival in colon cancer patients from the TCGA database. Table S6: The association of TMEM211/MMP2 or MMP9 co-expression with disease-specific survival in colon cancer patients from the TCGA database.
