# Supplementary material for: TMEM211 Promotes Tumor Progression and Metastasis in Colon Cancer
Source: Curr Issues Mol Biol. 2023 May 24;45(6):4529–43. doi: 10.3390/cimb45060287 (PMC10297151; doi:10.3390/cimb45060287)

Supplementary figures

Figure S1. TMEM211 expression and migration in normal colon FHC cells and different colorectal cancer cells.

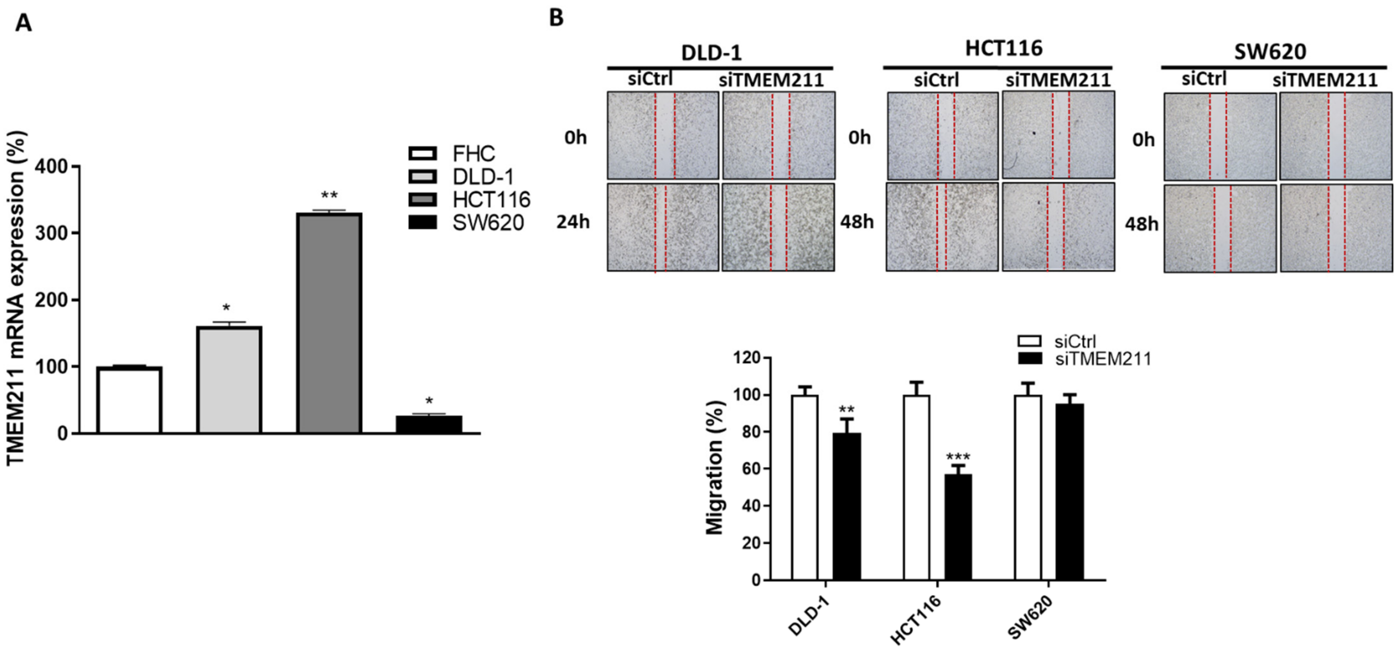

Figure S2. The protein levels of E-cad, N-cad and Snail in TMEM211-silenced DLD-1 cells were analyzed by Western blotting.

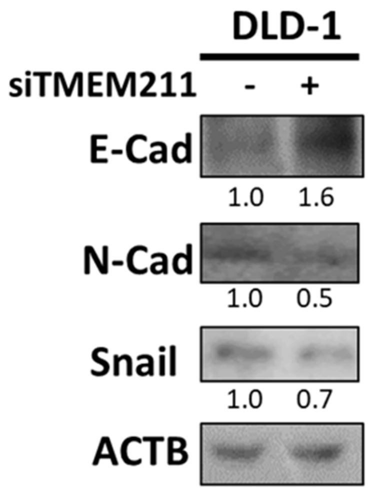

Figure S3. The gene expression levels of RelA and Akt2 in TMEM211-silenced DLD-1 cells were analyzed by RT-PCR.

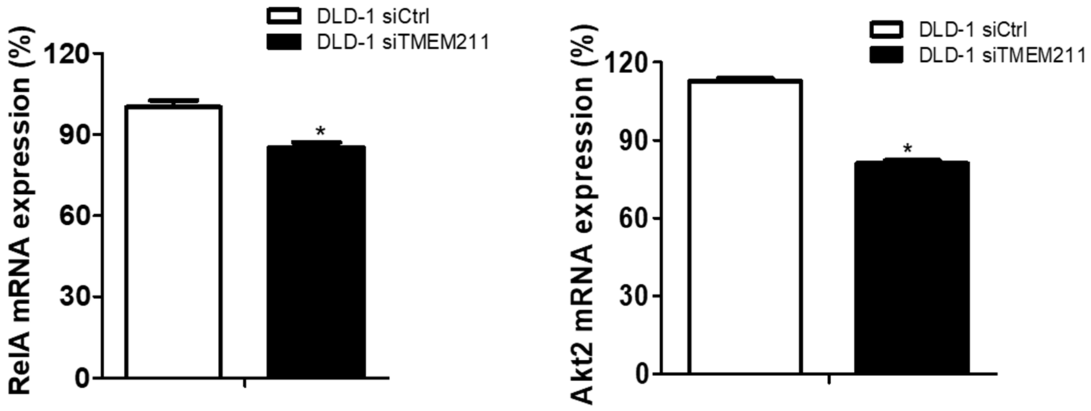

Figure S4. The MMP9 activity of TMEM211-silenced DLD-1 cells was analyzed by zymography.

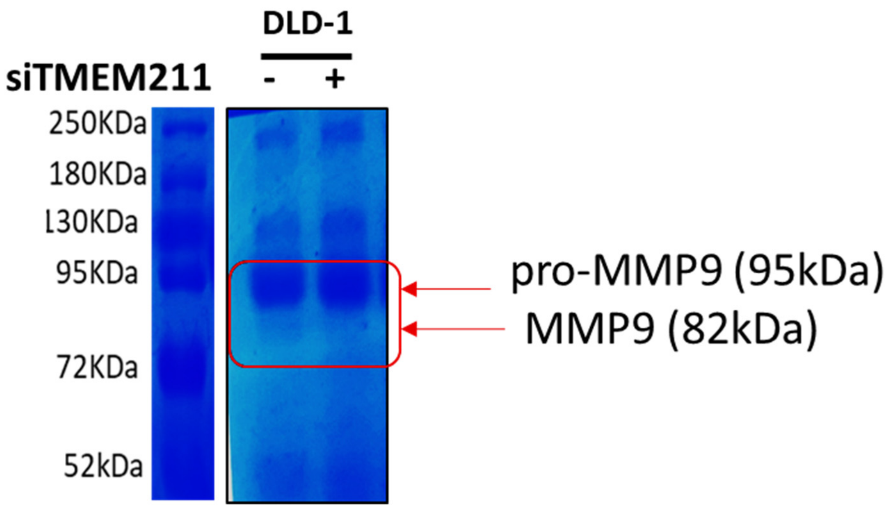

Supplement: Supplementary file 1 [file cimb-45-00287-s001.zip › supplementary figures (20230523).pdf]
