# Supplementary material for: TMEM211 Promotes Tumor Progression and Metastasis in Colon Cancer
Source: Curr Issues Mol Biol. 2023 May 24;45(6):4529–43. doi: 10.3390/cimb45060287 (PMC10297151; doi:10.3390/cimb45060287)
Supplement: Supplementary file 1 [file cimb-45-00287-s001.zip › supplementary tables (20230523).pdf]

Supplementary tables

Table S1. AJCC/UICC-TNM and clinical staging for colon cancer patients.

AJCC stage	TNM stage			TNM criteria
	T	N	M	
0	*in situ*	0	0	Tumor confined to mucosa
I	1	0	0	Tumor invades into submucosa
I	2	0	0	Tumor invades into muscularis propria
IIA	3	0	0	Tumor invades into subserosa
IIB	4	0	0	Tumor invades into adjacent organs
IIIA	1-2	1	0	Tumor metastases to 1-3 nodes
IIIB	3-4	1	0	Tumor metastases to 1-3 nodes
IIIC	1-4	2	0	Tumor metastases to 4 or more nodes
IV	1-4	0-2	1	Metastases to distant sites

Table S2. The association of TMEM211 expression with clinicopathologic outcomes in colon cancer patients from TCGA database.

Variable	No. (%)	Mean±SD	Median	*p value**
Sex				
Female	122 (45.0)	3.75 ± 2.03	3.9116	0.405
Male	149 (55.0)	3.96 ± 2.06	4.2920	
Age, yrs				
≦70	161 (59.4)	3.90 ± 2.08	4.0863	0.744
＞70	110 (40.6)	3.82 ± 2.01	4.0342	
AJCC pathological stage				
I + II	152 (56.1)	3.98 ± 2.01	4.1238	0.294
III + IV	119 (43.9)	3.72 ± 2.10	3.9192	
T classification				
T1 + T2	47 (17.3)	3.83 ± 2.09	4.0863	0.886
T3 + T4	224 (82.7)	3.87 ± 2.05	4.0632	
N classification				
N0	157 (57.9)	3.95 ± 2.00	4.1196	0.429
N1 +N2	114 (42.1)	3.75 ± 2.11	3.9236	

Abbreviations: AJCC, American Joint Committee on Cancer

*p values were estimated by student's t-test.

Table S3. The association of TMEM211 expression with overall survival colon cancer patients from TCGA database.

Variable	ROC	No. (%)	CHR (95% CI)	*p value*[*]	AHR (95% CI)	*p value*[†]
Sex						
Female	Low	98 (80.3)	1		1	
	High	24 (19.7)	0.99 (0.37−2.67)	0.990	1.04 (0.39−2.78)	0.944[a]
Male	Low	115 (77.2)			1	
	High	34 (22.8)	1.73 (0.90−3.34)	0.101	2.21 (1.12−4.40)	0.023[a]
Age, yrs						
≦ 70	Low	125 (77.6)	1		1	
	High	36 (22.4)	1.28 (0.56−2.94)	0.562	1.85 (0.79−4.31)	0.154[a]
＞70	Low	88 (80.0)	1		1	
	High	22 (20.0)	1.64 (0.80−3.36)	0.177	1.78 (0.86−3.66)	0.119[a]
AJCC pathological stage						
I, II	Low	118 (77.6)	1			
	High	34 (22.4)	1.29 (0.55−3.02)	0.563	ND	ND
III, IV	Low	95 (79.8)	1			
	High	24 (20.2)	1.81(0.90−3.65)	0.096	ND	ND
T classification						
T1, T2	Low	36 (76.6)	1		1	
	High	11 (23.4)	7.49 (0.75−74.86)	0.086	5.99 (0.57−63.22)	0.137[b]
T3, T4	Low	177 (79.0)	1		1	
	High	47 (21.0)	1.29 (0.73−2.29)	0.381	1.43 (0.80−2.56)	0.222[b]
N classification						
N0	Low	123 (78.3)	1		1	
	High	34 (21.7)	1.21 (0.53−2.80)	0.653	1.21 (0.52−2.78)	0.659[c]
N1, N2	Low	90 (78.9)	1		1	
	High	24 (21.1)	1.83 (0.90−3.71)	0.094	1.81 (0.89−3.69)	0.104[c]

Abbreviations: ROC, operator characteristic curve for low and high expression; CHR, crude hazard ratio; CI, confidence interval; AHR, adjusted hazard ratio.

[*]p values were estimated by Cox's regression.

[†]p values were estimated by multivariate Cox's regression.

[a]Adjusted for AJCC pathological stage (stage III + IV vs stage I + II).

[b]Adjusted for N classification (N1, N2 vs N0).

[c]Adjusted for T classification (T3, T4 vs T1 + T2).

ND: Non-determined.

Table S4. The association of TMEM211 expression with progression-free interval survival in colon cancer patients from TCGA database.

Variable	ROC	No. (%)	CHR (95% CI)	*p value*[*]	AHR (95% CI)	*p value*[†]
Sex						
Female	Low	43 (35.2)	1		1	
	High	79 (64.8)	1.85 (0.75−4.52)	0.180	1.88 (0.77−4.61)	0.167[a]
Male	Low	46 (30.9)	1		1	
	High	103 (69.1)	1.72 (0.88−3.37)	0.116	2.00 (1.01−3.96)	0.048[a]
Age, yrs						
≦ 70	Low	52 (32.3)	1		1	
	High	109 (67.7)	1.13 (0.60−2.12)	0.698	1.26 (0.67−2.37)	0.478[a]
＞70	Low	37 (33.6)	1		1	
	High	73 (66.4)	4.53 (1.38−14.86)	0.013	4.98 (1.51−16.47)	0.008[a]
AJCC pathological stage						
I, II	Low	47 (30.9)	1			
	High	105 (69.1)	2.60 (0.90−7.44)	0.077	ND	ND
III, IV	Low	42 (35.3)	1			
	High	77 (64.7)	1.58 (0.83−3.00)	0.162	ND	ND
T classification						
T1, T2	Low	17 (36.2)	1		1	
	High	30 (63.8)	39.37 (0.016−94736.14)	0.355	305856.10 (0.00−4.258E + 293)	0.970[b]
T3, T4	Low	72 (32.1)	1		1	
	High	152 (67.9)	1.54 (0.89−2.64)	0.121	1.65 (0.96−2.85)	0.071[b]
N classification						
N0	Low	49 (31,2)	1		1	
	High	108 (68.8)	2.32 (0.89−6.02)	0.084	2.21 (0.85−5.73)	0.105[c]
N1, N2	Low	40 (35.1)	1		1	
	High	74 (64.9)	1.57 (0.81−3.06)	0.183	1.57 (0.81−3.06)	0.185[c]

Abbreviations: ROC, operator characteristic curve for low and high expression; CHR, crude hazard ratio; CI, confidence interval; AHR, adjusted hazard ratio.

[*]p values were estimated by Cox's regression.

[†]p values were estimated by multivariate Cox's regression.

[a]Adjusted for AJCC pathological stage (stage III + IV vs stage I + II).

[b]Adjusted for N classification (N1, N2 vs N0).

[c]Adjusted for T classification (T3, T4 vs T1 + T2).

ND: Non-determined.

Table S5. The association of TMEM211 expression with disease-free interval survival in colon cancer patients from TCGA database.

**Variable**	**ROC**	**No. (%)**	**CHR (95% CI)**	***p value***[*]	**AHR (95% CI)**	***p value***[†]
Sex						
Female	Low	11 (27.5)	1		1	
	High	29 (72.5)	1.80 (0.20–16.17)	0.601	1.95 (0.21–18.06)	0.559[a]
Male	Low	16 (29.1)	1		1	
	High	39 (70.9)	35.580 (0.09–14764.69)	0.246	293605.72 (0.00–3.632E+240)	0.964[a]
Age, yrs						
≦ 70	Low	19 (30.2)	1		1	
	High	44 (69.8)	3.58 (0.44–29.24)	0.233	3.69 (0.45–30.20)	0.224[a]
＞70	Low	8 (25.0)	1		1	
	High	24 (75.0)	33.820 (0.02–66199.71)	0.363	289457.37 (0.00–1.817E+295)	0.971[a]
AJCC pathological stage						
I, II	Low	18 (26.5)	1			
	High	50 (73.5)	34.52 (0.08–15191.10)	0.254	ND	ND
III, IV	Low	9 (33.3)	1			
	High	18 (66.7)	2.68 (0.30–24.26)	0.381	ND	ND
T classification						
T1, T2	Low	7 (28.0)	1		1	
	High	18 (72.0)	35.83 (0.00–11423601.49)	0.5<br>80	35.83 (0.00–11423601.49)	0.580[b]
T3, T4	Low	20 (28.6)	1		1	
	High	50 (71.4)	5.46 (0.70–42.54)	0.105	5.55 (0.71–43.28)	0.102[b]
N classification						
N0	Low	18 (26.9)	1		1	
	High	49 (73.1)	34.95 (0.06–21421.73)	0.278	287034.83 (0.00–4.833e+255)	0.966[c]
N1, N2	Low	9 (32.1)	1		1	
	High	19 (67.9)	3.19 (0.37–27.57)	0.293	3.19 (0.37–27.57)	0.293[c]

Abbreviations: ROC, operator characteristic curve for low and high expression; CHR, crude hazard ratio; CI, confidence interval; AHR, adjusted hazard ratio.

[*]p values were estimated by univariate Cox's regression.

[†]p values were estimated by multivariate Cox's regression.

[a]Adjusted for AJCC pathological stage (stage III + IV vs stage I + II).

[b]Adjusted for N classification (N1, N2 vs N0).

[c]Adjusted for T classification (T3, T4 vs T1 + T2).

ND: Non-determined.

Table S6. The association of TMEM211/MMP2 or MMP9 co-expression with disease-specific survival in colon cancer patients from TCGA database.

Variable	ROC	No. (%)	CHR (95% CI)	*p value*[*]	AHR (95% CI)	*p value*[†]
TMEM211	Low	139 (54.3)	1.00		1.00	
	High	117 (45.7)	1.81 (0.91−3.59)	0.090	2.30 (1.15−4.60)	0.019[a]
MMP2	Low	92 (35.9)	1.00		1.00	
	High	164 (64.1)	2.13 (0.96−4.71)	0.062	1.93 (0.87−4.28)	0.105[a]
TMEM211 (L), MMP2 (L)		47 (18.4)	1.00		1.00	
TMEM211 (H), MMP2 (L)		45 (17.6)	0.91 (0.38−2.20)	0.832	3.23 (0.65−16.03)	0.151[b]
TMEM211 (L), MMP2 (H)		92 (35.9)	1.00 (0.49−2.02)	0.995	3.48 (0.78−15.58)	0.102[b]
TMEM211 (H), MMP2(H)		72 (28.1)	2.10 (1.06−4.18)	0.034	5.76 (1.31−25.42)	0.021[b]
MMP9	Low	154 (35.5)	1.00		1.00	
	High	102 (64.5)	1.48 (0.76−2.92)	0.251	1.32 (0.67−2.61)	0.416[a]
TMEM211 (L), MMP9 (L)		78 (30.5)	1.00		1.00	
TMEM211 (H), MMP9 (L)		76 (29.7)	1.41 (0.71−2.83)	0.326	2.75 (0.98−7.74)	0.055[b]
TMEM211 (L), MMP9 (H)		61 (23.8)	1.16 (0.54−2.48)	0.708	2.44 (0.82−7.31)	0.110[b]
TMEM211 (H), MMP9(H)		41 (16.0)	1.63 (0.71−3.76)	0.251	3.30 (1.04−10.45)	0.042[b]

Abbreviations: ROC, operator characteristic curve for low and high expression; CHR, crude hazard ratio; CI, confidence interval; AHR, adjusted hazard ratio.

[*]p values were estimated by univariate Cox's regression.

†p values were estimated by multivariate Cox's regression.

[a]p values were adjusted for cell differentiation (moderate + poor vs. well) and AJCC pathological stage (stage III + IV vs stage I + II) by multivariate Cox's regression.

[b]p values were adjusted for group comparison by multivariate Cox's regression.
